# Evaluating the prognostic value of radiomics and clinical features in metastatic prostate cancer using [^68^Ga]Ga-PSMA-11 PET/CT

**DOI:** 10.1007/s13246-024-01516-8

**Published:** 2025-01-09

**Authors:** Kaylee Molin, Nathaniel Barry, Suki Gill, Ghulam Mubashar Hassan, Roslyn J. Francis, Jeremy S. L. Ong, Martin A. Ebert, Jake Kendrick

**Affiliations:** 1 School of Physics, Mathematics and Computing, University of Western Australia, Crawley, WA Australia; 2 Centre for Advanced Technologies in Cancer Research (CATCR), Perth, WA Australia; 3 Department of Radiation Oncology, Sir Charles Gairdner Hospital, Nedlands, WA Australia; 4 School of Allied Health, University of Western Australia, Crawley, WA Australia; 5 Department of Nuclear Medicine, Sir Charles Gairdner Hospital, Nedlands, WA Australia; 6 Medical School, University of Western Australia, Crawley, WA Australia; 7 Department of Nuclear Medicine, Fiona Stanley Hospital, Murdoch, WA Australia

**Keywords:** Radiomics, PSMA PET, Prostate cancer, Prognostic biomarkers

## Abstract

Prostate cancer is a significant global health issue due to its high incidence and poor outcomes in metastatic disease. This study aims to develop models predicting overall survival for patients with metastatic biochemically recurrent prostate cancer, potentially helping to identify high-risk patients and enabling more tailored treatment options. A multi-centre cohort of 180 such patients underwent [^68^Ga]Ga-PSMA-11 PET/CT scans, with lesions semi-automatically segmented and radiomic features extracted from lesions. The analysis included two phases: univariable and multivariable. Univariable analysis used Kaplan–Meier curves and Cox proportional hazards models to correlate individual features with overall survival. Multivariable analysis used the LASSO Cox proportional hazards method to create 13 models: radiomics-only, clinical-only, and various combinations of radiomic and clinical features. Each model included six features and was bootstrapped 1000 times to obtain concordance indices with 95% confidence intervals, followed by optimism correction. In the univariable analysis, 6 out of 8 clinical features and 68 out of 89 radiomic features were significantly correlated with overall survival, including age, disease stage, total lesional uptake and total lesional volume. The optimism-corrected concordance indices from the multivariable models were 0.722 (95% CI 0.653–0.784) for the clinical model, 0.681 (95% CI 0.616–0.745) for the radiomics model, and 0.704 (95% CI 0.648–0.768) for the combined model with three clinical and three radiomic features, when extracting radiomic features from the largest lesion only. While univariable analysis showed significant prognostic value for many radiomic features, their integration into multivariable models did not improve predictive accuracy beyond clinical features alone.

**Supplementary Information:**

The online version contains supplementary material available at 10.1007/s13246-024-01516-8.

## Introduction

Prostate cancer (PCa) is a major global health concern, ranking among the top cancers diagnosed in men and contributing significantly to cancer-related mortality. It represents about 29% of all male cancer diagnoses and is the second leading cause of cancer death [1]. While early-stage PCa often responds well to treatments such as radical prostatectomy or radiotherapy, a substantial proportion of patients—between 20 and 40%—experience biochemical recurrence (BCR) following treatment for localised disease [2, 3]. BCR is marked by rising prostate-specific antigen (PSA) levels after initial treatment, significantly increasing the risk of developing metastatic disease, which reduces the 5-year survival rate to only 30% [4]. Accurate identification of patients at higher risk of poor outcomes is thus important for optimising therapeutic strategies and improving prognosis. However, clinical management of PCa is complicated by disease complexity. PCa is heterogeneous from a clinical, morphological, and molecular perspective, often presenting multifocal tumours with distinct genomic and phenotypic characteristics within a single patient, making diagnosis, prognosis, and treatment difficult [5, 6]. Traditionally, clinical features such as age, Gleason score, extent of disease and PSA levels have been used to stratify patients’ risk levels to aid in treatment management [7–9].

Recent advancements in imaging technologies and the field of radiomics—whereby a large number of quantitative features are extracted from medical images—offer promising avenues for enhancing prognostic accuracy and for better patient risk stratification [10]. Radiomics can capture subtle tumour characteristics and biomarkers, providing valuable diagnostic or prognostic information, with the potential to aid clinical decision-making [10, 11]. The central hypothesis is that these extracted features reflect the underlying biology of the tumour, which can guide personalised therapy [12]. This approach enables objective characterisation of tumours, overcoming the variability of visual assessments by radiologists [13, 14]. Additionally, radiomics is non-invasive, and can capture intra-tumoral heterogeneity affecting disease progression and response [15, 16]. By incorporating radiomics into conventional clinical workflows, clinicians may be able to make more informed decisions regarding the most appropriate treatment strategies for individual patients, further optimising outcomes and minimising unnecessary interventions. However, challenges such as variability in the radiomics workflow and the need for scientific rigor must be addressed to ensure reproducibility and clinical translation [17, 18].

Among the imaging modalities for PCa, [^68^Ga]Ga-Prostate Specific Membrane Antigen (PSMA)−11 PET/CT has emerged as a highly sensitive and specific tool for detecting PCa lesions, especially in the context of BCR disease [19, 20]. This imaging technique targets PSMA which is a transmembrane protein that is overexpressed in malignant prostate tissues, enabling detailed visualisation of recurrent and metastatic lesions [21]. Over 90% of PCa cases overexpress PSMA, allowing [^68^Ga]Ga-PSMA-11 PET/CT to accurately target and visualise malignant deposits. Studies have shown that [^68^Ga]Ga-PSMA-11 PET/CT has higher detection rates for recurrent disease compared to conventional imaging methods, such as CT, MRI, and bone scintigraphy, even in patients with low PSA levels [19, 22–24]. This imaging modality can reliably stage PCa at presentation, aiding in optimal treatment planning. Additionally, it improves biopsy accuracy and guides surgery and radiotherapy [20], significantly impacting clinical management decisions.

This study explores the potential of integrating radiomic features from [^68^Ga]Ga-PSMA-11 PET scans with traditional clinical features to predict overall survival (OS) in patients with BCR metastatic PCa (mPCa). By developing and comparing predictive models, the study aims to determine whether combining these radiomic features with clinical data enhances prognostic accuracy. This approach is novel in its specific application of [^68^Ga]Ga-PSMA-11 PET/CT scans within a BCR mPCa cohort. The primary objectives are to (i) assess the correlation between radiomic features and OS in BCR mPCa patients through univariable analysis, and (ii) compare the predictive accuracy of clinical-only, radiomic-only, and combined feature models through multivariable models.

## Methods

### Patient cohort

The patient cohort for this retrospective study comprised 238 individuals diagnosed with BCR PCa. These patients were enrolled in a prospective trial registered with the Australian and New Zealand Clinical Trials Registry (ACTRN12615000608561) [24] and underwent imaging at either Sir Charles Gairdner Hospital or Fiona Stanley Hospital in Perth, Western Australia. To be included, patients had to meet specific criteria: firstly, they had to exhibit BCR after primary therapy, as indicated by serum PSA levels greater than 0.2 ng/mL at more than 6 weeks post radical prostatectomy or a PSA level 2 ng/mL higher than the previous nadir measurement at 3 months post external beam radiotherapy. Secondly, patients had to exhibit either oligometastatic disease (with three or fewer lesions) or negative disease on abdominopelvic contrast CT and bone scintigraphy.

Ethics approval for this study was granted by the Human Research Ethics Committees of Sir Charles Gairdner Hospital (RGS1736), Fiona Stanley Hospital (2015 − 125), and the University of Western Australia (2019/RA/4/20/6382). The study complied with the principles outlined in the Declaration of Helsinki.

### Scan acquisition

The imaging modality used for patient assessments was the [^68^Ga]Ga-PSMA-11 PET/CT scan. Patients underwent these scans 60 min after receiving an injection of 2 MBq/kg of [^68^Ga]Ga-PSMA-11. Scans were performed using either a Siemens Biograph 64 or Siemens Biograph 128 PET/CT scanner (CTI Inc, Knoxville, TN). Before imaging, patients were instructed to empty their bladder. A low-dose CT acquisition (50 mAs, 120 kVp) was then conducted for attenuation correction, followed by PET data acquisition. PET images were reconstructed to a pixel size of 4.07 × 4.07 mm^2^ in the axial plane, while CT images were reconstructed to pixel sizes of either 0.98 × 0.98 mm^2^ or 1.52 × 1.52 mm^2^. Both the CT and PET images had a slice thickness of 2 mm. Additionally, the PET scanners underwent quantitative consistency evaluation and were accredited under the Australasian Radiopharmaceutical Trials Network (ARTnet) scheme [25].

### Lesion delineation

Patient scans were evaluated and segmented by an experienced nuclear medicine physician (JO). Using the E-PSMA 5-point scoring system, areas with increased radiotracer uptake were identified as lesions if they were classified as ‘definitely’ or ‘probably’ positive [26]. Other areas were deemed negative and excluded. A semi-automated delineation method was used, initially applying a threshold of 3 standardised uptake value (SUV) units normalised by body weight to the PET image. The segmentation was then manually adjusted to refine the delineation. The MIM Encore software (MIM Software Inc., Cleveland, OH, USA) was used for the delineation process.

### Feature extraction

Radiomic features were extracted from the PET images using Python (v3.9.1) and PyRadiomics (v3.1.0) [27], in accordance with the guidelines established by the Imaging Biomarker Standardisation Initiative (IBSI) [28]. Any deviations from the IBSI definitions are documented. The extracted features comprised first-order features, shape features, and texture features, with no image filters applied before extraction. A fixed intensity bin size of 1.14 SUV was used, resulting in an overall average of 64 bins across all patient scans. Studies have shown that using a fixed bin width improves the reproducibility of radiomic features in PET images [29], and hence, it is used as the default method in PyRadiomics.

In addition to the automatically extracted features, several radiomic features were manually calculated and included: total lesional volume (TLV), lesional bone volume, lesional volume within pelvic lymph nodes and lesional volume within distant lymph nodes. In addition, total lesional uptake (TLU) and total lesional quotient (TLQ) were calculated using the methods described by Seifert et al. [30]. These features were included based on previous literature suggesting their prognostic potential [30–32]. The definitions for pelvic and distant lesion volumes were defined according to Eiber et al. [33]. Although not explicitly defined, lesions in the inguinal nodes were also classified as pelvic due to their proximity within the pelvic region. Lesional liver volume was not included as a category as there were no liver metastases observed in the patient cohort. A total of 118 radiomic features were collected for each lesion in every patient, with a cumulative count of 735 lesions across included patients.

To convert lesion-level radiomic data into patient-level radiomic data, three methods were explored, resulting in three separate datasets [34, 35]. The first dataset comprised radiomic features from only the largest lesion within each patient. The second dataset included radiomic features from only the hottest lesion, defined as the lesion with the highest tracer uptake, as determined by the maximal SUV on the PET scans. The final dataset was derived from a weighted average of the three largest lesions by volume. Clinical features were then added to each of the datasets.

The clinical features recorded for each patient were weight, age, number of lesions, conventional staging (based on bone scan and abdominal-pelvic CT), staging based on PSMA PET/CT scan, PSA level at referral, Gleason score, and whether they underwent prostatectomy or radiotherapy treatment as initial treatment. Seven patients had missing data for Gleason score which was addressed using k-nearest-neighbours imputation with three neighbours. The outcome to predict was OS, defined by the time from a patient’s first scan to their death by any cause at the end of the follow-up period in March 2023.

### Feature selection

To enhance predictive performance and reduce model complexity, feature selection is used to identify the most influential variables from the extensive pool of radiomic features. This process strategically prunes irrelevant or redundant attributes, facilitating the creation of more robust and interpretable predictive models. Feature selection was used in the multivariable analysis.

The first method for feature selection was based on a prior study by Kendrick et al., which assessed the repeatability of radiomic features across test-retest scans for [^68^Ga]Ga-PSMA-11 PET/CT [36]. Radiomic features were evaluated using an Intraclass Correlation Coefficient (ICC) with 95% confidence intervals (CIs). Using these results, only features with a lower bound of the ICC 95% CI exceeding the ‘excellent’ threshold of 0.9 were included, reducing the initial 118 features to 89. This approach ensured that only highly repeatable features with low measurement variability were used in the modelling process, minimising the risk of overfitting.

Secondly, the Least Absolute Shrinkage and Selection Operator (LASSO) was used to perform variable selection and regularisation. LASSO helps in selecting a subset of predictors by imposing a penalty, thereby improving the model’s accuracy and interpretability by preventing overfitting [37].

### Sample size calculation

To determine an appropriate number of features to include in each model, a tool developed by van Smeden et al. [38] was used (https://mvansmeden.shinyapps.io/BeyondEPV/). By inputting an events fraction of 0.32 (57 deaths out of 180 patients) and a relative mean prediction squared error (rMPSE) of 10%, the tool indicated that only 150 samples were required for six candidate predictors. Additionally, when aiming for an rMPSE of 9%, the minimum sample size for six candidate predictors increased to exactly 180. As a result, six features were used in each model.

### Statistical analysis

**Fig. 1:**
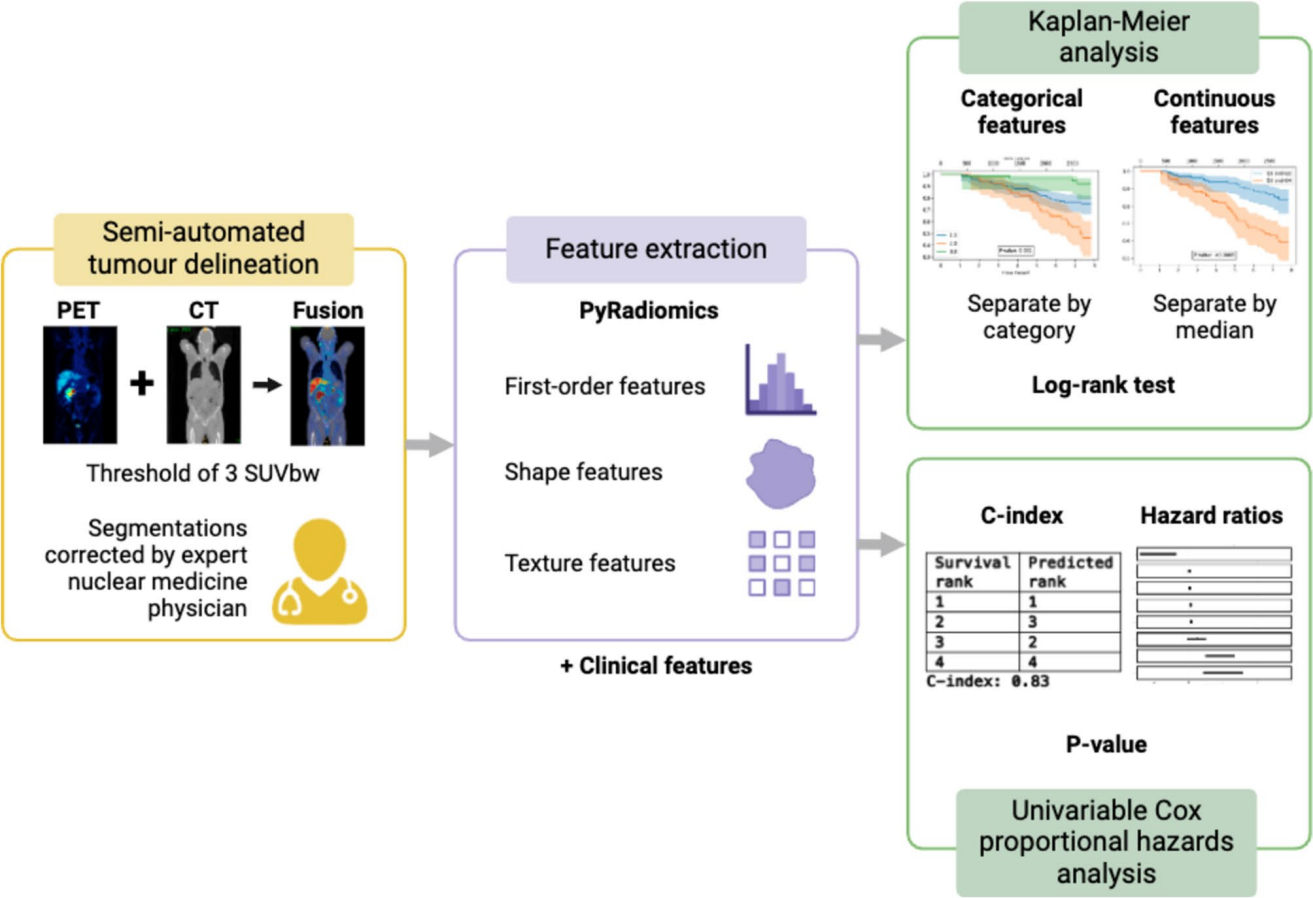
Workflow for univariable analysis. First, the regions of uptake greater than 3 standardised uptake values (SUV) normalised by body weight were automatically segmented and corrected by an experienced nuclear medicine physician. Radiomic features were then extracted from the largest lesion, hottest lesion, and weighted average of the largest three lesions, and combined with the patient’s clinical features. Univariable analysis was performed using Kaplan–Meier curves and the log-rank test. Categorical features were separated by group, and continuous features were dichotomised based on the median value. A univariable Cox proportional hazards analysis was also conducted to determine the hazard ratios, concordance indices, and significant features, based on a p-value of less than 0.05. PET, positron emission tomography; CT, computed tomography; SUVbw, standardised uptake values normalised by body weight; C-index, concordance-index

The statistical analysis for this study comprised two separate parts: univariable analysis and multivariable analysis. In the univariable analysis, each feature’s correlation with survival time was assessed separately using Kaplan–Meier curves and the Cox proportional hazards model. Categorical variables were plotted using Kaplan–Meier curves for each group, while continuous variables were dichotomised based on their median values. Differences in OS between the groups were evaluated using the log-rank test. Volume data (TLV, TLQ, TLU, lesional bone volume, lesional pelvic lymph node volume, lesional distant lymph node volume) was transformed by adding one and taking the natural logarithm [32]. Figure 1 shows this workflow.

The Cox proportional hazards model was used to estimate hazard ratios (HRs) with their corresponding 95% CIs and p-values. Prediction accuracy was evaluated using Harrell’s concordance index (C-index) for discrimination.

**Fig. 2:**
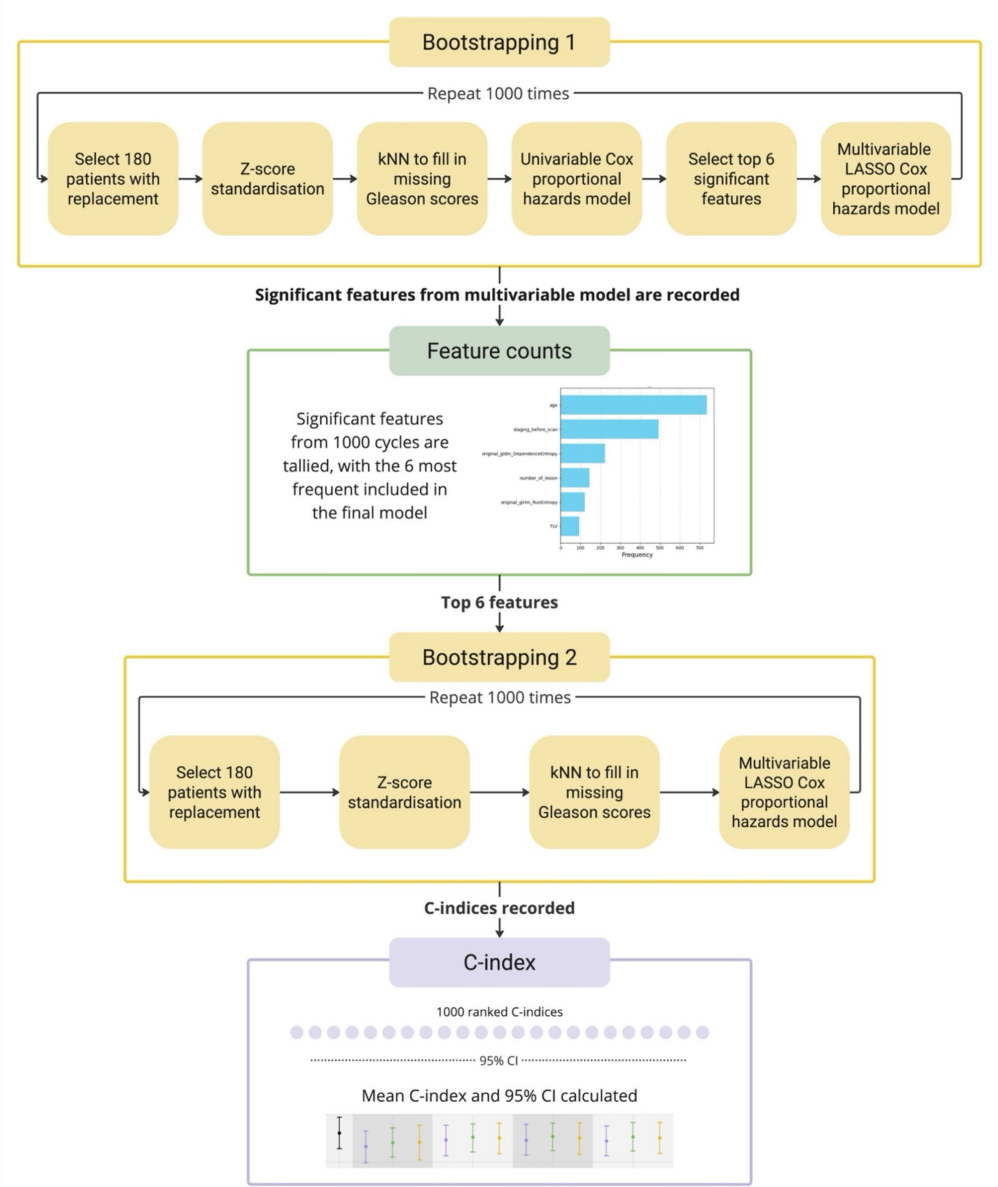
Pipeline for multivariable analysis. Each of the 13 models underwent this process. The initial round of bootstrapping identifies prognostic features. The top 6 features are then input into the next round of bootstrapping to calculate the concordance-indices with 95% confidence intervals. C-indices, concordance indices; kNN, k-nearest neighbours; LASSO, least absolute shrinkage and selection operator; CI, confidence interval

A comprehensive pipeline for multivariable analysis was developed to compare statistical models, as seen in Fig. 2. The three datasets (largest lesion, hottest lesion, and weighted average of the largest three lesions) each underwent the entire pipeline to evaluate how different lesions correlate with survival outcomes, resulting in a total of 13 models. These models were: a clinical-feature-only model, radiomic-feature-only models for each of the three datasets, and combination models with varying numbers of clinical and radiomic features, specifically: three clinical and three radiomic features, four clinical and two radiomic features, and five clinical features with one radiomic feature, each tested across the three datasets.

The analysis consisted of two parts: ‘bootstrapping 1’ and ‘bootstrapping 2’. In ‘bootstrapping 1’, internal validation was performed using 1000 bootstrapped samples from the original 180-patient dataset. This involved resampling with replacement to create datasets of equal size. Within each cycle, z-score standardisation and data imputation using k-nearest neighbours were applied. A univariable Cox proportional hazards model was then used to select the top six features using the smallest p-values for input into a multivariable LASSO Cox proportional hazards model [37]. Features with p-values less than 0.05 in the multivariable LASSO Cox model were considered significant and were recorded.

After 1000 bootstrapping repetitions, the frequency of significant features was noted. The six features most frequently significant were then used in ‘bootstrapping 2’ to reduce variability and improve the C-indices by selecting known prognostic features. In this phase, the dataset was again bootstrapped, with data imputation and z-score standardisation performed within each cycle. The data was input into a final multivariable LASSO Cox proportional hazards model using the six significant features from ‘bootstrapping 1’. For each of the 1000 cycles, C-indices were recorded. These C-indices were optimism-corrected [39] and ranked, with the 25^th^ and 975^th^ values used to establish 95% CIs for each of the 13 models. Each model was then compared based on the 95% CIs for the optimism-corrected C-indices.

All statistical analysis was conducted in Python using statistical analysis packages, including lifelines (v0.28.0) for survival analysis [40], and NumPy and pandas for data manipulation and processing.

## Results

### Patient characteristics

Out of the initial 238 patients retrospectively enrolled in the study, 48 (20%) did not have metastases and 10 (4%) had metastases that comprised only a single voxel, making them unsuitable for radiomic feature extraction. They were consequently excluded from the analysis, leaving 180 patients for further investigation. 57 (32%) out of 180 patients had died by the last follow-up. The median survival time was not reached as more than half of the patients were still alive at the end of the follow-up period. The median follow-up time was 2705 days (range, 391–2857) calculated using the reverse Kaplan–Meier method. Patient characteristics can be found in Table 1.

**Table 1:** Patient characteristics

Characteristic	All patients (*n* = 180)
Age, years	70.3 (45.8–90.0)
Weight, kg	88.5 (60–153)
PSA level at referral, ng/mL	3.95 (0.2–79.46)
Number of lesions identified at baseline imaging	2 (1–35)
Conventional staging	
0	145 (80.6%)
1	35 (19.4%)
Staging based on PSMA PET/CT scan	
0	123 (68.3%)
1	51 (28.3%)
2	6 (3.3%)
Gleason score*	
< 8	96 (53.3%)
≥ 8	77 (42.8%)
Previous definitive treatment	
Prostatectomy	98 (54.4%)
Radiotherapy	82 (45.6%)
Administered treatment	
Active surveillance	26 (14.4%)
ADT	111 (61.7%)
Radiotherapy	65 (36.1%)
Chemotherapy	8 (4.4%)
Surgery	4 (2.2%)
Status	
Deaths	57 (31.7%)
Alive at study end	123 (68.3%)

*PSA,* prostate specific antigen; *ADT,* androgen deprivation therapy

*Data missing for 7 patients. Continuous data is reported as the median value with the range provided in parentheses, whereas nominal data is presented as the count along with the percentage of the total in parentheses

### Univariable analysis

**Fig. 3:**
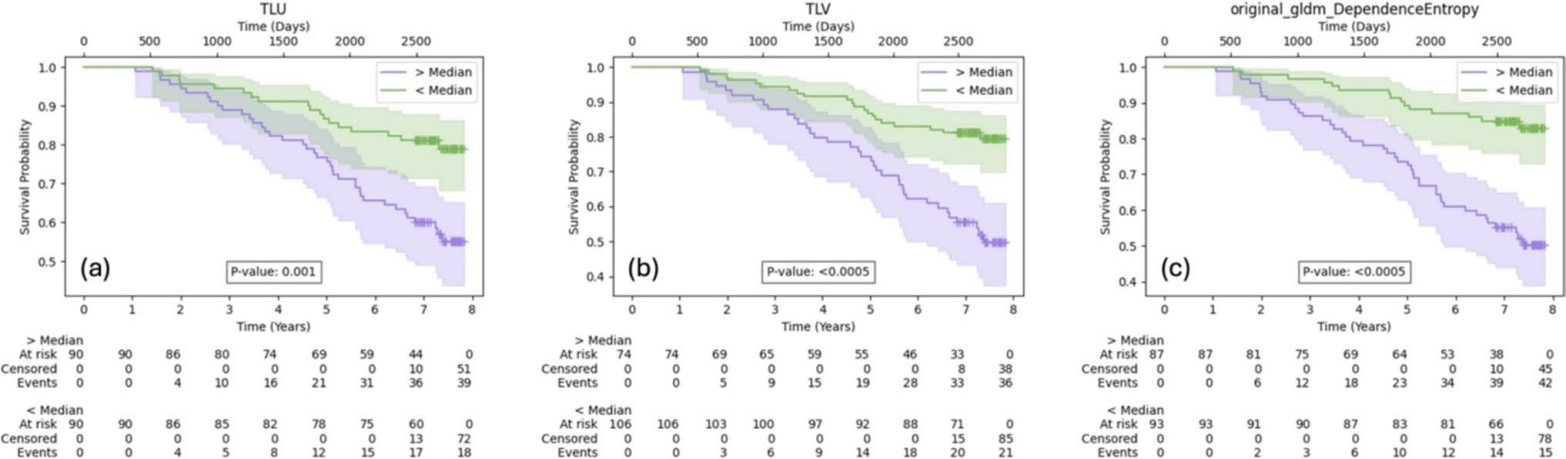
Kaplan–Meier curves for **a** TLU, total lesional uptake; **b** TLV, total lesional volume and **c** original_gldm_DependenceEntropy. Data was dichotomised by the median value. P-values from the log-rank test are displayed to assess the differences in survival between the two groups

For the univariable Cox proportional hazards models, 68 (78%) out of the 89 radiomic features were found to be correlated with survival time (p-value < 0.05) based on data from the largest lesion. Notably, the features with the highest C-indices were TLU (C-index = 0.707, p-value = $\:7.26\times\:{10}^{-8}$), TLV (C-index = 0.704, p-value = $\:2.16\times\:{10}^{-7}$) and original_gldm_DependenceEntropy (C-index = 0.704, p-value = $\:3.12\times\:{10}^{-7}$). The corresponding Kaplan–Meier curves for these features are found in Fig. 3 and the full data is available in Online Resource 1 (Supplementary Table 1).

**Fig. 4:**
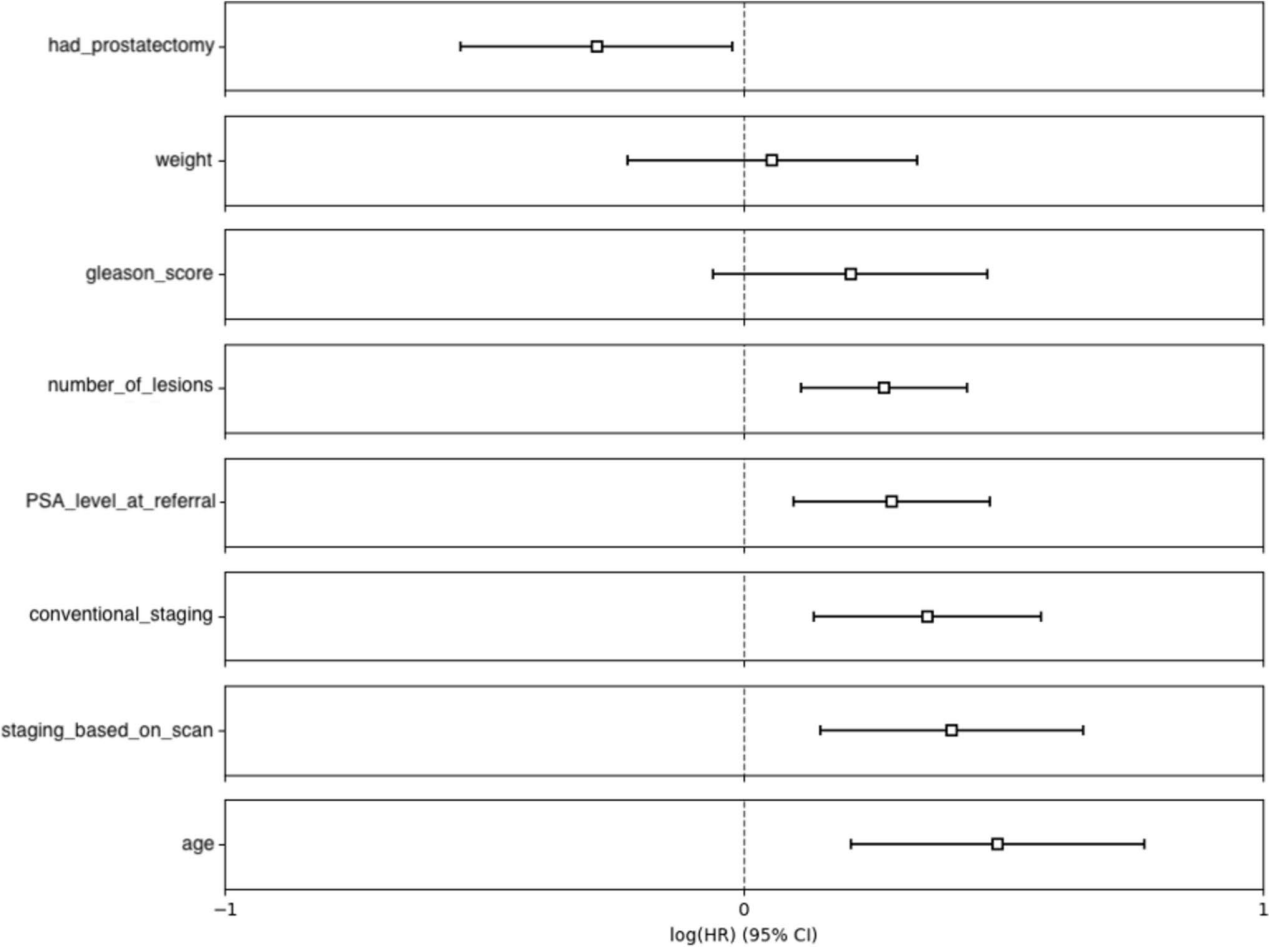
Natural logarithm of the hazard ratios for clinical features, ordered from smallest to largest. All features are significant except for weight and Gleason score

For the clinical features, 6 out of 8 (75%) were significantly correlated with survival time when applying the Cox proportional hazards model, with weight and Gleason score being the non-significant features. Figure 4 shows the natural logarithm of the HRs for clinical features. Numerical data for the HRs and the corresponding upper and lower bounds of the 95% CIs, p-values and C-indices are presented in Online Resource 1 (Supplementary Table 2). Notably, the number of lesions that a patient had was associated with the highest C-index of 0.654 (p-value = $\:8.34\times\:{10}^{-4}$) with a corresponding HR of 1.31 (95% CI 1.12–1.54), followed by age with a C-index of 0.615 (p-value=$\:6.84\times\:{10}^{-4}$) and a corresponding HR of 1.63 (95% CI 1.23–2.17).

In the Kaplan–Meier analysis, 5 (63%) out of 8 clinical features were associated with OS when dichotomising the data by median value, while 81 (91%) out of 89 radiomic features were significantly correlated. All Kaplan–Meier data is found in Online Resource 1 (Supplementary Tables 3 and 4).

### Multivariable analysis

**Fig. 5:**
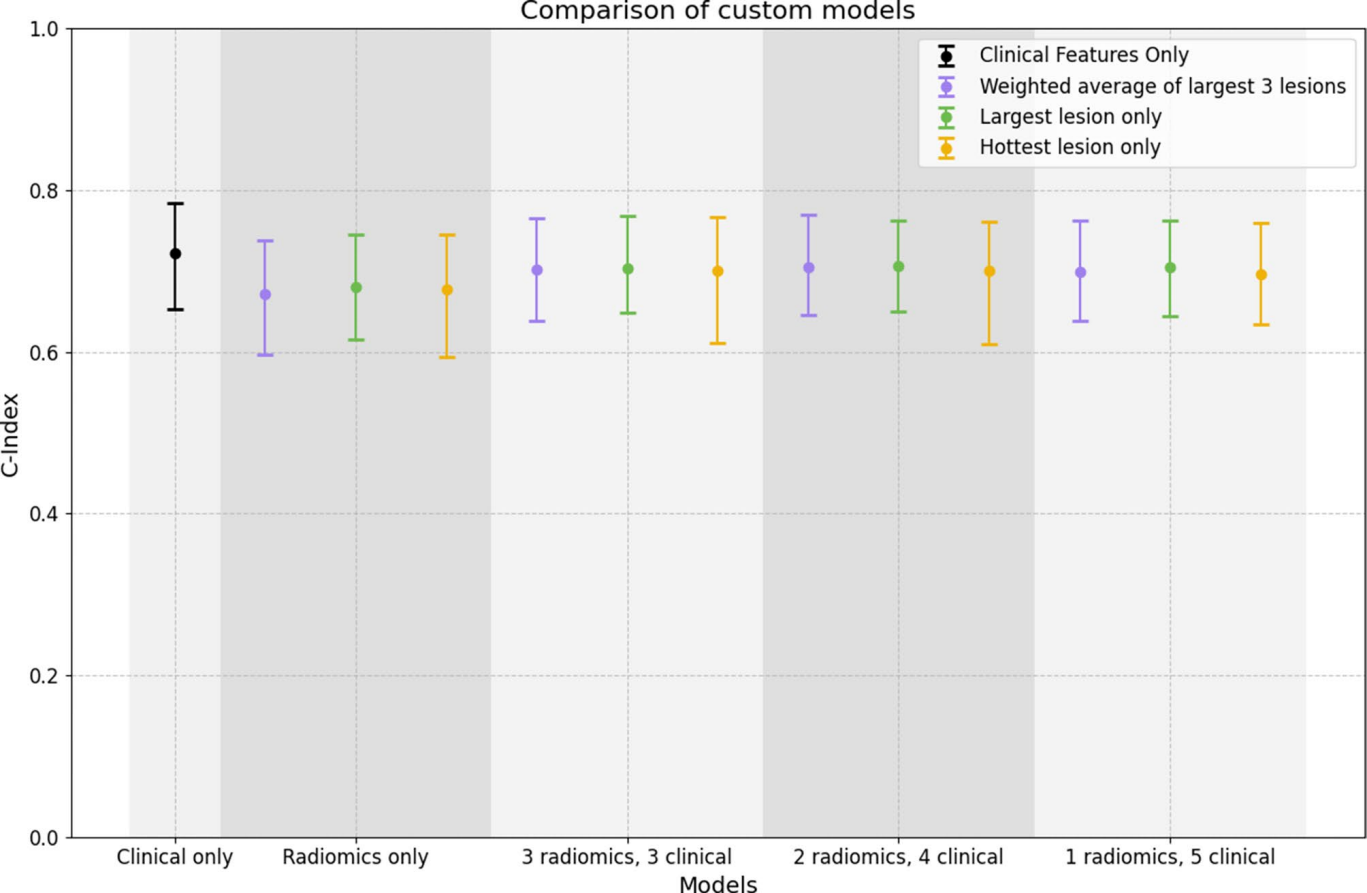
C-indices with 95% confidence intervals for 13 models using the top 6 significant features from prior analysis

The results of the final optimised bootstrapped models using the top six prognostic features for each model type are shown in Fig. 5, and Table 2 contains the features used in creating each model. The clinical model demonstrated the highest prognostic performance, achieving a C-index of 0.722 (95% CI 0.653–0.784), outperforming all radiomic and combination models. The combination model incorporating four clinical features (‘conventional_staging’, ‘age’, ‘gleason_score’, ‘number_of_lesions’) and two radiomic features (‘original_gldm_DependenceEntropy’, ‘original_shape_MajorAxisLength’) based on the largest tumour data followed, with a C-index of 0.706 (95% CI 0.65–0.762). In contrast, the radiomic-only model, which used the weighted average of the three largest lesions, had the lowest C-index at 0.671 (95% CI 0.596–0.738).

**Table 2:** Features used in each of the 13 Cox proportional hazard models and the corresponding C-indices with 95% confidence intervals

Model name	Features used in model	C-index
Clinical	‘conventional_staging’, ‘age’, ‘gleason_score’, ‘number_of_lesions’, ‘staging_based_on_scan’, ‘had_prostatectomy’	0.722 (95% CI 0.653–0.784)
Radiomics only—weighted average	‘original_gldm_DependenceEntropy’, ‘original_shape_Sphericity’, ‘original_shape_MajorAxisLength’, ‘original_glrlm_RunEntropy’, ‘TLU’, ‘original_glcm_DifferenceEntropy’	0.671 (95% CI 0.596–0.738)
Radiomics only—largest	‘original_gldm_DependenceEntropy’, ‘original_shape_MajorAxisLength’, ‘original_glrlm_RunEntropy’, ‘original_shape_Sphericity’, ‘original_shape_Maximum3DDiameter’, ‘original_glcm_DifferenceEntropy’	0.681 (95% CI 0.616–0.745)
Radiomics only—hottest	‘original_glszm_ZoneEntropy’, ‘original_ngtdm_Contrast’, ‘TLU’, ‘original_glrlm_RunEntropy’, ‘TLQ’, ‘original_glcm_DifferenceAverage’	0.678 (95% CI 0.594–0.745)
3 clinical, 3 radiomics—weighted average	‘conventional_staging’, ‘age’, ‘gleason_score’, ‘original_gldm_DependenceEntropy’, ‘original_shape_Sphericity’, ‘original_shape_MajorAxisLength’	0.702 (95% CI 0.639–0.765)
3 clinical, 3 radiomics—largest	‘conventional_staging’, ‘age’, ‘gleason_score’, ‘original_gldm_DependenceEntropy’, ‘original_shape_MajorAxisLength’, ‘original_glrlm_RunEntropy’	0.704 (95% CI 0.648–0.768)
3 clinical, 3 radiomics—hottest	‘conventional_staging’, ‘age’, ‘gleason_score’, ‘original_glszm_ZoneEntropy’, ‘original_ngtdm_Contrast’, ‘TLU’	0.700 (95% CI 0.611–0.767)
4 clinical, 2 radiomics—weighted average	‘conventional_staging’, ‘age’, ‘gleason_score’, ‘number_of_lesions’, ‘original_gldm_DependenceEntropy’, ‘original_shape_Sphericity’	0.705 (95% CI 0.645–0.77)
4 clinical, 2 radiomics—largest	‘conventional_staging’, ‘age’, ‘gleason_score’, ‘number_of_lesions’, ‘original_gldm_DependenceEntropy’, ‘original_shape_MajorAxisLength’	0.706 (95% CI 0.65–0.762)
4 clinical, 2 radiomics—hottest	‘conventional_staging’, ‘age’, ‘gleason_score’, ‘number_of_lesions’, ‘original_glszm_ZoneEntropy’, ‘original_ngtdm_Contrast’	0.701 (95% CI 0.61–0.761)
5 clinical, 1 radiomics—weighted average	‘conventional_staging’, ‘age’, ‘gleason_score’, ‘number_of_lesions’, ‘staging_based_on_scan’, ‘original_gldm_DependenceEntropy’	0.699 (95% CI 0.638–0.762)
5 clinical, 1 radiomics—largest	‘conventional_staging’, ‘age’, ‘gleason_score’, ‘number_of_lesions’, ‘staging_based_on_scan’, ‘original_gldm_DependenceEntropy’	0.705 (95% CI 0.644–0.762)
5 clinical, 1 radiomics—hottest	‘conventional_staging’, ‘age’, ‘gleason_score’, ‘staging_based_on_scan’, ‘number_of_lesions’, ‘original_glszm_ZoneEntropy’	0.696 (95% CI 0.634–0.759)

The clinical features that were most frequently prognostic, in decreasing order, were conventional staging, age, Gleason score, number of lesions, staging based on PSMA PET/CT scan, and prostatectomy as opposed to radiotherapy in initial treatment. For the radiomic features, the most prognostic features were ‘original_gldm_DependenceEntropy’, ‘original_shape_Sphericity’, ‘original_shape_MajorAxisLength’, ‘original_glrlm_RunEntropy’, ‘TLU’ and ‘original_glcm_DifferenceEntropy’.

## Discussion

In this study, predictive models were developed for OS in patients with BCR mPCa using [^68^Ga]Ga-PSMA-11 PET/CT images. To our knowledge, this is the first study to evaluate and compare the accuracy of clinical, radiomic, and combination models in predicting OS for these patients using [^68^Ga]Ga-PSMA-11 PET scans. Our results indicate that the clinical-only, radiomics-only, and combination feature models performed similarly when evaluated by the C-index in the multivariable analysis, with the clinical-only model outperforming the others. Sound statistical techniques were employed throughout the research, including the use of repeatable radiomic features, LASSO for feature selection and regularisation, and bootstrapping to obtain the C-indices and 95% CIs. Additionally, optimism correction was used for internal validation by evaluating all available patient data, instead of relying on a hold-out test set.

The variable nature of radiomic features and their reliance on precise tumour delineation could have contributed to the observed lack of performance improvement with radiomic models. Variability in tumour segmentation can introduce inconsistencies, as the manual or semi-automated delineation of tumour boundaries is subject to observer variability [31]. Additionally, variations in imaging acquisition settings, such as scanner calibration, image resolution, and reconstruction algorithms, can significantly impact radiomic features. However, the PET scanners used in this study were accredited under the ARTnet scheme, which aimed to minimise variability by ensuring quantitative harmonisation across scanners.

Furthermore, while radiomics is intended to capture the underlying biological characteristics of tumours, it is possible that some features reflect imaging artifacts or methodological limitations rather than true tumour biology. The heterogeneity of tumour biology within individual patients, specifically in PCa, can also mean that radiomic features derived from a single or few lesions might not represent the entire disease burden. This inherent variability may also explain why the clinical-feature-only model outperformed the combination model. The clinical features included were likely already strong predictors, and replacing some of these with radiomic features, which are more variable and less consistent, may have diluted the overall predictive power. Conversely, clinical features such as age and PSA levels are well-established prognostic factors with known correlations to outcomes in PCa. These factors are consistently measured and less susceptible to the technical variations that affect radiomic features.

The extraction of radiomic features from medical images has gained popularity as a non-invasive method to gather comprehensive patient information. While many anticipate that radiomics will aid in diagnosis, prognosis [41, 42] and the path to personalised cancer care, it is important to approach this advancement with caution. The radiomics pipeline, from image acquisition to predictive modelling using radiomic features, is highly variable and lacks the necessary standardisation, including feature extraction and selection processes. This further complicates the validation of these studies. This variability is reflected in this study’s results, where the clinical-feature-only model outperformed all models incorporating radiomic features, with the radiomic-feature-only model performing the poorest.

Although radiomic features did not improve the multivariable survival models, the univariable analysis revealed significant correlations between many radiomic features and OS. Specifically, 68 (76%) out of 89 radiomic features were significantly correlated with OS using a Cox proportional hazards model, with TLU, TLV, and original_gldm_DependenceEntropy showing the highest C-indices. TLU and TLV likely ranked highest because they quantify key characteristics of tumour burden, such as PSMA uptake and total volume, which are closely associated with disease progression and OS in mPCa. The relationship between original_gldm_DependenceEntropy and tumour characteristics is less straightforward, as it is a higher-order texture feature. However, it may capture characteristics of the tumour, such as heterogeneity and complexity, that are not measurable using traditional techniques, potentially providing additional prognostic insights. These features could form the basis of a clinical decision-support tool, offering clinicians more comprehensive information to guide personalised treatment planning. Additionally, 6 (75%) out of 8 clinical features (prostatectomy vs. radiotherapy, PSA level at referral, number of lesions, age, staging based on PSMA PET/CT scan and conventional staging) demonstrated prognostic potential. These features may be useful for stratifying patients into high and low-risk groups; however, further research is required to confirm their utility.

Many other studies have investigated more advanced PCa cases. For instance, Gafita et al. developed models combining clinical and imaging features to predict outcomes after ^177^Lu-PSMA therapy in an advanced disease cohort, achieving a C-index of 0.71 (95% CI 0.69–0.73) for the OS model, which closely matches our combination model results for an earlier disease cohort [43]. Acar et al. found that PSMA tumour volume was prognostic via univariable analysis, supported in our study by the ‘total lesional volume’ feature [44]. Moazemi et al. identified minimum SUV and kurtosis as the most important variables among all radiomic features [45]. In contrast, our analysis found that while kurtosis was prognostic in both the Cox proportional hazards model and Kaplan–Meier analysis, minimum SUV was not. This discrepancy may be due to different disease burdens and treatment regimes between the studies, and the small sample size in Moazemi’s study. Gutsche et al. extracted radiomic features from [^68^Ga]Ga-PSMA-11 PET scans for predicting OS, achieving a C-index of 0.67 with combined radiomic and clinical parameters, outperforming models using radiomics or clinical features alone [42].

An additional challenge in radiomic analyses is converting lesion-level data to patient-level data. We compared three aggregation methods: the largest lesion, the ‘hottest’ lesion identified by maximal tracer uptake (based on maximal SUV), and a weighted average of the three largest lesions by volume. Carlier et al. found that features from the largest lesion had superior predictive capabilities in diffuse large B-cell lymphoma patients [34]. Chang et al. reported that a weighted average of the three largest lesions had the highest C-index in patients with multiple brain metastases [35]. Our study found comparable performance across the three methods, with the largest lesion showing a slight but non-significant advantage.

In addition to the feature selection methods described, Minimum Redundancy Maximum Relevance (mRMR) [46, 47] was also trialled with the expectation that it might reduce computational time and improve predictive performance. However, this method provided little improvement in predictive accuracy and required substantial computational time, hence it was excluded from the final analysis.

Certain limitations should be noted in this study. First, the retrospective nature and relatively small sample size limit the generalisability of our findings. Although data from two centres were used, the sample size from the second centre was insufficient for validation [48]. Sample size calculations were used to determine the optimum number of features to minimise error, but overfitting remains a risk [48, 49]. Riley et al. assert that the optimal sample size is context-specific and depends on various factors, including the total number of participants, the incidence rate, the expected model performance, and the ratio of events to candidate predictors [48]. To address these considerations, they developed a tool in R and Strata called ‘pmsampsize’. Using this tool with our dataset, a significantly larger sample size is recommended to mitigate the risk of overfitting. Unfortunately, due to the retrospective nature of the study, we were unable to include more participants. Consequently, it should be acknowledged that there is a possibility that our results, using six candidate predictors may suffer from overfitting.

Additionally, the Cox proportional hazards model assumes proportional hazards over time, and violations of this assumption in our univariable analysis may bias HRs and affect interpretability. However, this assumption is less critical for our predictive objectives using multivariable models. Even with a large sample size, minor violations of proportional hazards can occur, and many datasets inherently violate this assumption. According to Stensrud et al., strictly fitting proportional hazards can change the scientific question and slightly reduce power [50]. Future work should explore nonlinear models for survival prediction, such as random survival forests and support vector machines, to better capture relationships that may not be identified using linear methods.

Managing the high dimensionality of radiomic features is another challenge. Effective feature selection remains difficult, and unsupervised clustering techniques may help identify candidate predictors prior to further supervised feature selection. Exploring such methods could enhance the stability and predictive power of developed radiomic models.

## Conclusion

This study explored the predictive power of clinical and radiomic features derived from [^68^Ga]Ga-PSMA-11 PET/CT scans in patients with BCR mPCa. While the univariable analysis indicated that many radiomic features had significant prognostic value, the integration of these features into multivariable models did not enhance predictive accuracy over clinical features alone. These findings suggest that the incorporation of radiomic features into routine clinical practice in BCR PCa requires further investigation.

## Supplementary Information

Below is the link to the electronic supplementary material.

Supplementary file1 (DOCX 40 kb)

## Data Availability

The datasets generated and/or analysed during the current study are not publicly available due to privacy and ethics restrictions but are available from the corresponding author on reasonable request. No personally identifying information will be disclosed.

## Abbreviations

ARTnet: Australasian Radiopharmaceutical Trials Network
BCR: Biochemical recurrence
CI: Confidence interval
CT: Computed tomography
HR: Hazard ratio
IBSI: Image biomarker standardisation initiative
ICC: Intraclass correlation coefficient
LASSO: Least absolute shrinkage and selection operator
mPCa: Metastatic prostate cancer
mRMR: Minimum redundancy maximum relevance
OS: Overall survival
PCa: Prostate cancer
PET: Positron emission tomography
PSA: Prostate-specific antigen
PSMA: Prostate-specific membrane antigen
rMPSE: Relative mean prediction squared error
SUV: Standardised uptake value
TLQ: Total lesional quotient
TLU: Total lesional uptake
TLV: Total lesional volume
