# Supplementary material for: Evaluating the prognostic value of radiomics and clinical features in metastatic prostate cancer using [68Ga]Ga-PSMA-11 PET/CT
Source: Phys Eng Sci Med. 2025 Jan 9;48(1):329–41. doi: 10.1007/s13246-024-01516-8 (PMC11996952; doi:10.1007/s13246-024-01516-8)
Supplement: Supplementary file 1 — Supplementary file1 (DOCX 40 kb) [file 13246_2024_1516_MOESM1_ESM.docx]

Evaluating the prognostic value of radiomics and clinical features in metastatic prostate cancer using [^68^Ga]Ga-PSMA-11 PET/CT

Kaylee Molin^1,2,3*^, Nathaniel Barry^1,2^, Suki Gill^3,4^, Ghulam Mubashar Hassan^1^, Roslyn J. Francis^5,6^, Jeremy S. L. Ong^7^, Martin A. Ebert^1,2,3^, Jake Kendrick^1,2^

^1^ School of Physics, Mathematics and Computing, University of Western Australia, Crawley, WA, Australia.

^2^ Centre for Advanced Technologies in Cancer Research (CATCR), Perth, WA, Australia.

^3^ Department of Radiation Oncology, Sir Charles Gairdner Hospital, Nedlands, WA, Australia.

^4^ School of Allied Health, University of Western Australia, Crawley, WA, Australia

^5^ Department of Nuclear Medicine, Sir Charles Gairdner Hospital, Nedlands, WA, Australia.

^6^ Medical School, University of Western Australia, Crawley, WA, Australia.

^7^ Department of Nuclear Medicine, Fiona Stanley Hospital, Murdoch, WA, Australia.

*Corresponding author. E-mail(s): [22734429@student.uwa.edu.au](mailto:22734429@student.uwa.edu.au?subject=Paper%20Enquiry)

# Supplementary Data

Supplementary Table 1 Results of the univariable Cox proportional hazards model for radiomic features extracted from the largest lesion, ordered by ascending p-value.

| Feature | log(HR) | HR | HR 95% CI Lower Bound | HR 95% CI Upper Bound | P-value | C-index |
| --- | --- | --- | --- | --- | --- | --- |
| original_firstorder_TotalEnergy | 3.64694889 | 38.35745453 | 22.19697917 | 66.28353825 | 5.04233108E-39 | 0.6811957 |
| original_firstorder_Energy | 3.64731498 | 38.37149942 | 22.20381383 | 66.31166964 | 5.04758997E-39 | 0.68163746 |
| original_shape_MajorAxisLength | 0.57089432 | 1.76984915 | 1.4553334 | 2.15233569 | 1.07292221E-08 | 0.69047268 |
| original_shape_Maximum2DDiameterRow | 0.58208378 | 1.78976402 | 1.46089869 | 2.19266078 | 1.91921032E-08 | 0.69268149 |
| original_shape_Maximum3DDiameter | 0.59132752 | 1.80638484 | 1.46501658 | 2.22729643 | 3.14515582E-08 | 0.69393315 |
| original_glcm_DifferenceVariance | 0.93748906 | 2.55356153 | 1.82042172 | 3.58195928 | 5.65227533E-08 | 0.66919452 |
| original_glrlm_RunEntropy | 0.68367171 | 1.98113856 | 1.54423656 | 2.54165074 | 7.51736853E-08 | 0.68767486 |
| original_gldm_DependenceNonUniformity | 0.42850289 | 1.5349578 | 1.30416817 | 1.80658869 | 2.54399986E-07 | 0.68730673 |
| original_gldm_DependenceEntropy | 0.81379982 | 2.25646589 | 1.65204857 | 3.0820149 | 3.12440256E-07 | 0.70372552 |
| original_glcm_SumEntropy | 0.69905034 | 2.01184124 | 1.53852758 | 2.63076543 | 3.25440541E-07 | 0.68244736 |
| original_glszm_ZoneEntropy | 0.7304599 | 2.07603515 | 1.56160126 | 2.75993753 | 4.95972367E-07 | 0.68369901 |
| original_glcm_DifferenceEntropy | 0.63964924 | 1.89581579 | 1.47494582 | 2.43677933 | 5.90734003E-07 | 0.67258136 |
| original_glszm_GrayLevelNonUniformity | 0.35755514 | 1.42982941 | 1.24096372 | 1.6474391 | 7.54502484E-07 | 0.67287587 |
| original_glcm_JointEntropy | 0.6869055 | 1.98755552 | 1.51238449 | 2.61201895 | 8.32439354E-07 | 0.67479016 |
| original_shape_Maximum2DDiameterColumn | 0.53147462 | 1.70143944 | 1.37642973 | 2.10319213 | 8.92509398E-07 | 0.68627595 |
| original_firstorder_Entropy | 0.6304315 | 1.87842094 | 1.45599483 | 2.42340504 | 1.23159191E-06 | 0.66286261 |
| original_firstorder_Range | 0.32120081 | 1.37878242 | 1.20926127 | 1.57206802 | 1.59729069E-06 | 0.67854513 |
| original_glszm_SizeZoneNonUniformity | 0.36185215 | 1.43598661 | 1.23851252 | 1.66494688 | 1.63556107E-06 | 0.67125607 |
| original_firstorder_InterquartileRange | 0.30865649 | 1.36159457 | 1.19798709 | 1.54754572 | 2.29315825E-06 | 0.6713297 |
| original_glrlm_RunLengthNonUniformity | 0.33972628 | 1.40456307 | 1.21943308 | 1.61779884 | 2.46525304E-06 | 0.69091445 |
| TLU | 0.91674842 | 2.50114449 | 1.69818418 | 3.68377224 | 3.97633069E-06 | 0.70725961 |
| original_firstorder_MeanAbsoluteDeviation | 0.30249508 | 1.35323102 | 1.19000886 | 1.53884081 | 4.16373081E-06 | 0.67162421 |
| original_firstorder_Maximum | 0.31107689 | 1.36489417 | 1.19556752 | 1.55820232 | 4.26612500E-06 | 0.65572081 |
| original_firstorder_RobustMeanAbsoluteDeviation | 0.29993506 | 1.34977116 | 1.18777615 | 1.53385988 | 4.77085775E-06 | 0.673244 |
| original_glcm_DifferenceAverage | 0.36049395 | 1.43403759 | 1.22880583 | 1.67354658 | 7.95988177E-06 | 0.64798999 |
| original_shape_Maximum2DDiameterSlice | 0.43466008 | 1.54443798 | 1.27623557 | 1.86900345 | 1.04718874E-05 | 0.69356501 |
| original_glcm_JointAverage | 0.29353615 | 1.34116167 | 1.17703074 | 1.52817981 | 1.04718874E-05 | 0.66080106 |
| original_glcm_SumAverage | 0.29353615 | 1.34116167 | 1.17703074 | 1.52817981 | 1.31341872E-05 | 0.66080106 |
| original_shape_SurfaceArea | 0.36798277 | 1.44481715 | 1.22443686 | 1.70486258 | 2.89174378E-05 | 0.69857164 |
| original_shape_MinorAxisLength | 0.40515031 | 1.49952788 | 1.24018687 | 1.81310085 | 3.07552825E-05 | 0.68016492 |
| original_firstorder_90Percentile | 0.28095613 | 1.3243955 | 1.16047888 | 1.51146519 | 3.97512734E-05 | 0.63967015 |
| original_shape_SurfaceVolumeRatio | -0.83487206 | 0.43392999 | 0.29138559 | 0.64620642 | 6.61011090E-05 | 0.6811957 |
| original_gldm_LargeDependenceHighGrayLevelEmphasis | 0.28937106 | 1.33558722 | 1.15861222 | 1.53959468 | 7.27650507E-05 | 0.6595494 |
| original_shape_LeastAxisLength | 0.4028613 | 1.49609936 | 1.22608204 | 1.825582 | 9.21453043E-05 | 0.6869386 |
| original_firstorder_RootMeanSquared | 0.27646806 | 1.31846484 | 1.14785736 | 1.51442992 | 1.10347269E-04 | 0.62509203 |
| original_shape_Sphericity | -0.48398456 | 0.61632272 | 0.48224059 | 0.78768504 | 1.91882512E-04 | 0.60447651 |
| original_firstorder_Mean | 0.270789 | 1.31099841 | 1.13709884 | 1.51149292 | 2.54315577E-04 | 0.61728759 |
| distant_lymph_volume | 0.3943472 | 1.4834155 | 1.20247609 | 1.82999193 | 2.68524312E-04 | 0.60550729 |
| original_glcm_Idm | -0.56578389 | 0.56791479 | 0.41939483 | 0.76903 | 7.06843210E-04 | 0.64077455 |
| original_glcm_Id | -0.56980439 | 0.56563607 | 0.41632585 | 0.7684946 | 9.61982927E-04 | 0.64136357 |
| original_firstorder_Median | 0.26075628 | 1.29791131 | 1.11612159 | 1.50931025 | 1.10486504E-03 | 0.59888087 |
| original_shape_VoxelVolume | 0.27096241 | 1.31122578 | 1.11638872 | 1.54006666 | 1.13992258E-03 | 0.69555294 |
| original_shape_MeshVolume | 0.26800839 | 1.30735811 | 1.11292793 | 1.53575553 | 1.17405533E-03 | 0.69562656 |
| original_firstorder_Uniformity | -0.62003482 | 0.53792571 | 0.37025836 | 0.78151934 | 1.38560859E-03 | 0.66124282 |
| original_glrlm_GrayLevelNonUniformityNormalized | -0.63892712 | 0.52785845 | 0.35886 | 0.77644358 | 1.56419622E-03 | 0.6625681 |
| original_glcm_Contrast | 0.21708159 | 1.24244547 | 1.08765403 | 1.41926634 | 1.66378093E-03 | 0.6524076 |
| original_glrlm_LongRunHighGrayLevelEmphasis | 0.86123403 | 2.3660787 | 1.38747193 | 4.03491292 | 1.70196622E-03 | 0.68752761 |
| original_glcm_ClusterTendency | 0.85623444 | 2.35427879 | 1.38063308 | 4.01455587 | 2.52102724E-03 | 0.67287587 |
| original_glcm_MaximumProbability | -0.61114439 | 0.54272942 | 0.37050868 | 0.79500221 | 3.30811556E-03 | 0.66890001 |
| original_firstorder_Kurtosis | 0.32881196 | 1.38931658 | 1.12240274 | 1.71970408 | 4.05907937E-03 | 0.61876012 |
| original_ngtdm_Complexity | 3.39830011 | 29.91320759 | 3.09851405 | 288.7835827 | 4.74370485E-03 | 0.66551318 |
| original_ngtdm_Contrast | 0.27386446 | 1.31503655 | 1.0909714 | 1.58512049 | 5.19717738E-03 | 0.61220733 |
| original_glcm_SumSquares | 0.89204297 | 2.44010962 | 1.31379278 | 4.53201988 | 5.28887712E-03 | 0.67066706 |
| pelvic_lymph_volume | 1.21297883 | 3.36348901 | 1.43997684 | 7.85641683 | 5.29768623E-03 | 0.68377886 |
| original_glszm_HighGrayLevelZoneEmphasis | 0.18933741 | 1.20844863 | 1.0581745 | 1.38006357 | 5.30740081E-03 | 0.66433515 |
| original_gldm_GrayLevelVariance | 0.18930197 | 1.20840579 | 1.05787823 | 1.38035223 | 5.38682668E-03 | 0.66617582 |
| original_firstorder_Variance | 0.18926435 | 1.20836035 | 1.0578392 | 1.38029932 | 5.53817509E-03 | 0.67206597 |
| original_glrlm_GrayLevelVariance | 0.18924844 | 1.20834112 | 1.05780424 | 1.38030101 | 6.25041517E-03 | 0.6663967 |
| original_glszm_GrayLevelVariance | 0.1887878 | 1.20778463 | 1.05741665 | 1.37953542 | 6.32353685E-03 | 0.66794287 |
| original_glcm_Autocorrelation | 0.18916813 | 1.20824408 | 1.05707845 | 1.38102687 | 6.62343006E-03 | 0.66183184 |
| original_glrlm_HighGrayLevelRunEmphasis | 0.18712577 | 1.20577892 | 1.05442448 | 1.37885911 | 0.01200755 | 0.66109557 |
| original_gldm_HighGrayLevelEmphasis | 0.18699763 | 1.20562442 | 1.05418792 | 1.37881512 | 0.02818815 | 0.66021205 |
| original_glrlm_ShortRunHighGrayLevelEmphasis | 0.18628777 | 1.2047689 | 1.05318248 | 1.37817342 | 0.03134546 | 0.65424827 |
| original_gldm_SmallDependenceEmphasis | 0.30002554 | 1.34989329 | 1.06814794 | 1.70595459 | 0.04401300 | 0.59284347 |
| bone_volume | 1.36061025 | 3.89857169 | 1.20447407 | 12.61867037 | 0.05231785 | 0.54226182 |
| original_glcm_ClusterShade | 0.16334013 | 1.1774371 | 1.01762083 | 1.36235235 | 0.07338264 | 0.66904727 |
| original_glcm_InverseVariance | -0.28183177 | 0.75440059 | 0.58366076 | 0.97508739 | 0.08346736 | 0.61250184 |
| original_glcm_ClusterProminence | 0.15541581 | 1.16814359 | 1.00417605 | 1.35888467 | 0.10435659 | 0.67537918 |
| TLQ | 0.47043419 | 1.60068904 | 0.98095234 | 2.61195709 | 0.10788328 | 0.60769965 |
| original_glrlm_GrayLevelNonUniformity | 0.17093284 | 1.18641107 | 0.98394621 | 1.43053676 | 0.12737987 | 0.62236784 |
| original_glszm_ZonePercentage | 0.22267486 | 1.24941428 | 0.97096337 | 1.60771877 | 0.14619267 | 0.56243558 |
| original_glrlm_RunLengthNonUniformityNormalized | 0.2597835 | 1.29664933 | 0.94468338 | 1.77974919 | 0.18872483 | 0.59004565 |
| TLV | 0.44165727 | 1.55528261 | 0.85780527 | 2.81987542 | 0.20947760 | 0.61253809 |
| original_glrlm_ShortRunEmphasis | 0.2523511 | 1.28704784 | 0.91574146 | 1.80890812 | 0.23922891 | 0.60859962 |
| original_glrlm_RunPercentage | 0.22072366 | 1.24697879 | 0.89725278 | 1.73301899 | 0.33048296 | 0.59483139 |
| original_glszm_SizeZoneNonUniformityNormalized | -0.188431 | 0.82825765 | 0.60517816 | 1.13356823 | 0.35284327 | 0.55529377 |
| original_shape_Flatness | -0.12796791 | 0.87988162 | 0.67997262 | 1.138563 | 0.38765150 | 0.54645855 |
| original_gldm_DependenceNonUniformityNormalized | -0.14223055 | 0.86742125 | 0.64257364 | 1.17094692 | 0.48726743 | 0.53188043 |
| original_glszm_SmallAreaHighGrayLevelEmphasis | -1.41593761 | 0.24269795 | 0.00977048 | 6.02859866 | 0.59153139 | 0.35215727 |
| original_gldm_GrayLevelNonUniformity | 0.06856516 | 1.07097041 | 0.88259476 | 1.29955181 | 0.65944275 | 0.60263584 |
| original_glcm_Imc1 | 0.06333225 | 1.06538075 | 0.80385054 | 1.41199897 | 0.67526547 | 0.48159329 |
| original_glrlm_RunVariance | 0.04962636 | 1.05087837 | 0.83314367 | 1.32551611 | 0.69223855 | 0.44882933 |
| original_glszm_LargeAreaHighGrayLevelEmphasis | -0.07617068 | 0.92665803 | 0.63550042 | 1.35121092 | 0.71833535 | 0.36629362 |
| original_ngtdm_Busyness | -0.06540618 | 0.93668692 | 0.6565055 | 1.33644333 | 0.72253598 | 0.5216463 |
| original_glrlm_LongRunEmphasis | 0.03055038 | 1.03102184 | 0.78946706 | 1.3464856 | 0.82252004 | 0.40877632 |
| original_gldm_LargeDependenceEmphasis | -0.01995765 | 0.98024019 | 0.72715572 | 1.32140999 | 0.89579596 | 0.57281696 |
| original_glcm_Idn | 0.00470928 | 1.00472039 | 0.76529931 | 1.31904346 | 0.97294949 | 0.52032101 |

Supplementary Table 2 Results of the univariable Cox proportional hazards model for clinical features, ordered by ascending p-value. HR, hazard ratio; CI, confidence interval.

| Feature | log(HR) | HR | HR 95% CI Lower Bound | HR 95% CI Upper Bound | P-value | C-index |
| --- | --- | --- | --- | --- | --- | --- |
| Age | 0.49034514 | 1.6328797 | 1.23039776 | 2.16701965 | 0.00068409 | 0.61508267 |
| Number of lesions | 0.27072717 | 1.31091736 | 1.1184082 | 1.5365627 | 0.00083486 | 0.65433567 |
| Conventional staging | 0.35449598 | 1.42546201 | 1.14554228 | 1.7737817 | 0.0014822 | 0.57927917 |
| Staging based on PSMA PET/CT scan | 0.40095036 | 1.49324314 | 1.16045935 | 1.92145902 | 0.00182836 | 0.58695135 |
| PSA level at referral | 0.28538423 | 1.33027306 | 1.10192519 | 1.60594061 | 0.00297716 | 0.61121684 |
| Had prostatectomy (as opposed to RT) | -0.2829988 | 0.75352071 | 0.57974831 | 0.97937923 | 0.03436697 | 0.57327227 |
| Gleason score | 0.20593798 | 1.228677 | 0.94256012 | 1.60164548 | 0.1278592 | 0.54579256 |
| Weight | 0.05544198 | 1.05700769 | 0.79951892 | 1.39742189 | 0.69711581 | 0.49286309 |

Supplementary Table 3 Results of the Kaplan-Meier curve log-rank test for clinical features, ordered by descending p-value. RT, radiotherapy.

| Feature | P-Value |
| --- | --- |
| Conventional staging | 0.00102286 |
| Staging based on PSMA PET/CT scan | 0.00109163 |
| Number of lesions | 0.00719127 |
| Age | 0.0282366 |
| Had prostatectomy (as opposed to RT) | 0.03202321 |
| PSA level at referral | 0.07063149 |
| Gleason score | 0.13129951 |
| Weight | 0.96026076 |

Supplementary Table 4 Results of the Kaplan-Meier curve log-rank test for radiomic features, ordered by descending p-value.

| Feature | P-Value |
| --- | --- |
| original_glszm_SizeZoneNonUniformity | 6.44E-07 |
| original_glszm_ZoneEntropy | 1.87E-06 |
| original_glszm_HighGrayLevelZoneEmphasis | 3.70E-06 |
| original_gldm_DependenceNonUniformity | 4.90E-06 |
| original_glszm_GrayLevelNonUniformity | 4.96E-06 |
| original_gldm_DependenceEntropy | 5.64E-06 |
| original_shape_Maximum2DDiameterRow | 1.43E-05 |
| original_shape_SurfaceArea | 1.78E-05 |
| TLV | 7.59E-05 |
| original_shape_MeshVolume | 9.44E-05 |
| original_glrlm_RunLengthNonUniformity | 9.58E-05 |
| original_glszm_GrayLevelVariance | 0.00013312 |
| original_shape_LeastAxisLength | 0.00018284 |
| original_shape_MajorAxisLength | 0.00019004 |
| original_glrlm_RunEntropy | 0.00023765 |
| original_glrlm_ShortRunEmphasis | 0.00030266 |
| original_shape_MinorAxisLength | 0.00030703 |
| original_glcm_DifferenceEntropy | 0.00036905 |
| original_shape_SurfaceVolumeRatio | 0.00046971 |
| original_firstorder_TotalEnergy | 0.00053697 |
| original_firstorder_Energy | 0.00053697 |
| original_shape_VoxelVolume | 0.00069061 |
| original_glcm_Autocorrelation | 0.00081714 |
| original_glrlm_GrayLevelNonUniformityNormalized | 0.00083728 |
| pelvic_lymph_volume | 0.00084067 |
| original_glcm_Idm | 0.00086901 |
| original_glcm_Id | 0.00086901 |
| original_glcm_MaximumProbability | 0.00087952 |
| original_firstorder_Entropy | 0.00088003 |
| original_glrlm_LongRunHighGrayLevelEmphasis | 0.00090539 |
| original_firstorder_Uniformity | 0.00092945 |
| original_glcm_SumAverage | 0.00093965 |
| original_glcm_JointAverage | 0.00093965 |
| original_gldm_GrayLevelVariance | 0.00098356 |
| original_firstorder_90Percentile | 0.00122605 |
| original_glcm_DifferenceVariance | 0.00129158 |
| original_glcm_ClusterShade | 0.00137249 |
| TLU | 0.00137441 |
| original_shape_Maximum2DDiameterSlice | 0.00165304 |
| original_glcm_Contrast | 0.00185876 |
| original_glcm_DifferenceAverage | 0.00185876 |
| original_glcm_SumEntropy | 0.00196155 |
| original_glcm_JointEntropy | 0.00206326 |
| original_glcm_SumSquares | 0.00206531 |
| original_ngtdm_Complexity | 0.00206531 |
| original_glrlm_GrayLevelVariance | 0.00217444 |
| original_glszm_SmallAreaHighGrayLevelEmphasis | 0.0024707 |
| original_firstorder_RobustMeanAbsoluteDeviation | 0.0030136 |
| original_glrlm_HighGrayLevelRunEmphasis | 0.00315577 |
| original_firstorder_Maximum | 0.00329961 |
| original_gldm_HighGrayLevelEmphasis | 0.00359404 |
| original_glrlm_RunLengthNonUniformityNormalized | 0.00359896 |
| original_firstorder_RootMeanSquared | 0.00360923 |
| original_glrlm_LongRunEmphasis | 0.00367126 |
| original_shape_Maximum2DDiameterColumn | 0.00380007 |
| original_glcm_InverseVariance | 0.00402359 |
| original_firstorder_MeanAbsoluteDeviation | 0.00407806 |
| original_firstorder_InterquartileRange | 0.00426874 |
| distant_lymph_volume | 0.00478609 |
| original_glrlm_RunPercentage | 0.00647866 |
| original_glcm_ClusterTendency | 0.00659482 |
| original_gldm_SmallDependenceEmphasis | 0.00673322 |
| original_glcm_ClusterProminence | 0.00673847 |
| original_firstorder_Kurtosis | 0.0077632 |
| original_glrlm_ShortRunHighGrayLevelEmphasis | 0.00787507 |
| original_shape_Maximum3DDiameter | 0.00831214 |
| original_firstorder_Median | 0.00970503 |
| original_firstorder_Variance | 0.00982797 |
| original_firstorder_Range | 0.01024664 |
| original_firstorder_Mean | 0.01072049 |
| original_shape_Sphericity | 0.01086585 |
| original_gldm_LargeDependenceEmphasis | 0.01140803 |
| original_shape_Flatness | 0.01606816 |
| original_gldm_LargeDependenceHighGrayLevelEmphasis | 0.01786856 |
| original_gldm_SmallDependenceHighGrayLevelEmphasis | 0.02019011 |
| original_ngtdm_Contrast | 0.02137196 |
| original_ngtdm_Strength | 0.021594 |
| original_glrlm_RunVariance | 0.02188446 |
| TLQ | 0.0228845 |
| bone_volume | 0.03013239 |
| original_glrlm_GrayLevelNonUniformity | 0.03589587 |
| original_glszm_ZonePercentage | 0.08168728 |
| original_glszm_SizeZoneNonUniformityNormalized | 0.27738269 |
| original_glszm_LargeAreaHighGrayLevelEmphasis | 0.29028449 |
| original_gldm_GrayLevelNonUniformity | 0.34511347 |
| original_glcm_Imc1 | 0.51611159 |
| original_ngtdm_Busyness | 0.55068648 |
| original_gldm_DependenceNonUniformityNormalized | 0.66908318 |
| original_glcm_Idn | 0.96982603 |
